# *Alternaria alternata* induced inflammatory lung responses: a novel *in vivo* PK/PD model

**DOI:** 10.1186/1476-9255-10-S1-P10

**Published:** 2013-08-14

**Authors:** Malgorzata Gil, Michael Caniga, Joseph Eckman, Robbie McLeod, Lily Moy, Alan Wilhelm, Janice Woodhouse, Jie Zhang-Hoover, Milenko Cicmil

**Affiliations:** 1 Merck Research Laboratories, Boston, MA 02115, USA

## Rationale

Asthma is a heterogeneous disorder characterized by several physiologic and immunologic phenotypes. Common environmental allergens such as pollen, house dust mite and mold induce airway inflammation and exacerbate asthmatic symptoms. Traditional rodent models of asthma use multiple sensitizations and challenges with allergens such as OVA and HDM to induce asthma like responses. *Alternaria alternata* is a fungal allergen linked to the development of severe asthma [1]. This allergen is capable of eliciting robust immune responses in the lungs [2]. In the current study we evaluated a single intratracheal (i.t.) instillation of *Alternaria* to model immune responses in Brown Norway rats.

## Methods

Brown Norway rats are commonly used to study allergic asthma. In this study, animals were subjected to a single i.t. challenge with gradient doses of *Alternaria alternata*. A temporal profile was performed following *Alternaria* challenge. Inflammatory cell infiltration and cytokines (IL-5, and IL-13) were assessed in bronchoalveolar lavage fluid (BALF). Pharmacological profiling was conducted using oral dosing of cotricosteriods.

## Results

*Alternaria* induced dose and time- dependent recruitment of inflammatory cells in the lungs along with increased cytokine levels in BALF. Here we report, a time related infiltration of neutrophils in BALF. Oral dosing of corticosteroids administered prior to *Alternaria* instillation led to a dose-dependent attenuation of *Alternaria* induced airway inflammation.

## Conclusions

A single i.t. instillation of *Alternaria* induced significant inflammation in the lung. Preliminary profiling suggests that *Alternaria* challenge has the potential to be a robust and reliable PK/PD model to assess *in vivo* compound potency.
